# Virological efficacy and immunological recovery among Ethiopian HIV-1 infected adults and children

**DOI:** 10.1186/1471-2334-14-28

**Published:** 2014-01-14

**Authors:** Andargachew Mulu, Uwe Gerd Liebert, Melanie Maier

**Affiliations:** 1 Department of Microbiology, College of Medicine and Health Sciences, University of Gondar, Gondar, Ethiopia; 2 Institute of Virology, University of Leipzig, Leipzig, Germany

**Keywords:** Antiretroviral, HIV viral load, CD4 T cells, HIV drug resistance, Ethiopia

## Abstract

**Background:**

Introduction of antiretroviral therapy (ART) in sub-Saharan Africa was a hot debate due to many concerns about adherence, logistics and resistance. Currently, it has been significantly scaled up. However as the WHO clinico-immunological approaches for initiation and monitoring of ART in the region lacks viral load determination and drug resistance monitoring, HIV infected adults and children may be at risk for “unrecognized” virologic failure and the subsequent development of antiretroviral drug resistance. This study evaluates the virological efficacy and immunological recovery of HIV/AIDS patients under ART.

**Methods:**

Consecutive HIV-1 infected adults (N = 100) and children (N = 100) who have been receiving ART for up to 6 years at Gondar University Hospital, Ethiopia were enrolled following the WHO protocol for assessment of acquired drug resistance. Magnitude of viral suppression, genotypic drug resistance mutations and patterns of CD4^+^ T cell recovery were determined using standard virological and immunological methods.

**Results:**

Virological suppression (HIV RNA < 40 copies/ml) was observed in 82 and 87% of adults and children on a median time of 24 months on ART, respectively. Mutation K103N conferring resistance to non nucleoside reverse transcriptase inhibitors and thymidine analogue mutations (M41L, L210W) were found only in one adult and child patient, respectively. Median CD4^+^ T cell count has increased from baseline 124 to 266 (IQR: 203–306) and 345 (IQR: 17–1435) to 998 (IQR: 678–2205) cells/mm^3^ in adults and children respectively after 12 months of ART. Nevertheless, small but significant number of clinically asymptomatic adults (16%) and children (13%) had low level viraemia (HIV-1 RNA 41–1000 copies/ml).

**Conclusions:**

Majority of both adults (82%) and children (87%) who received ART showed high viral suppression and immunological recovery. This indicates that despite limited resources in the setting virological efficacy can be sustained for a substantial length of time and also enhance immunological recovery irrespective of age. However, the presence of drug resistance mutations and low level viraemia among clinically asymptomatic patients highlights the need for virological monitoring.

## Background

The provision of antiretroviral therapy (ART) has reduced mortality of people living with HIV-infection [1,2]. Conceivably, the use of ART especially in low in-come countries with sub optimal medication and patient’s follow-up may favour the emergence and transmission of drug resistant HIV-1 mutations. Particularly long term use of ART can lead to drug resistance especially among those individuals with previous mono- or dual-therapy or when temporary discontinuation in therapy has occurred [3]. This poses the risk that HIV with definite resistance profiles may be transmitted to uninfected people and thus will impair the success of ART programs. Moreover, introducing ART particularly to sub-Saharan Africa was debated controversially because of concerns about adherence and subsequent development of drug resistance, poor infrastructure, logistic and human capacity, and cost-effectiveness [4]. However, the World Health Organization (WHO) pilot ART feasibility study catalysed global efforts and ART has been significantly scaled up [5]. Nevertheless, long term challenges of providing ART will become increasingly evident, including late drug toxicities, treatment failure and emergence of drug resistance [3,6]. Good levels of adherence to ART in the first year of treatment [7,8] and a short-term virological efficacy comparable to industrialized countries [9] have been documented from some African countries. But, it has been shown that 10% of patients who commence ART develop some form of genotypic drug resistance after two years, and almost 30% of patients develop viral failure within six years after starting ART [10], thereby representing a threat to the control of transmitted multi drug resistance [11]. Studies from sub-Saharan Africa countries revealed a level of drug resistance as high as 50% [12-15]. Moreover, African patients with very low CD4^+^ T cell counts have a high risk of mortality both before and during the initial months of ART [16,17] and advanced pre-treatment immunodeficiency is also reported to be associated with reduced capacity for restoration of CD4^+^ T cell counts and its functional responses during ART [18-20]. This raises the concern that many patients entering ART programmes in sub-Saharan Africa may have limited potential for immune recovery. Despite the rapid scaling up of ART and its positive outcomes in adults, as of 2012 only 30% of HIV infected children (≤14 years) eligible for ART were receiving it. Moreover, the limited access of paediatric regimens, the challenges of paediatric ART adherence and the likelihood of HIV drug resistance development raise great public health concern about drug resistance in children receiving ART [21].

In Ethiopia HIV prevalence is estimated to be 1.5% among the general population with an estimate of about 800, 000 people living with HIV (78% adults and 22% children <14 years). By the end of 2012, there were 249,174 adults and 16,000 children on treatment, of whom 135 children and 865 adults were receiving second line drugs. However, in some regions 40% of patients who were enrolled to ART dropped out from treatment. The over all current ART coverage for adult populations is high (86% of estimated eligible). Conversely, the coverage of ART for children is low (only 20% of estimated eligible) (http://www.etharc.org/). Access to optimal laboratory monitoring with viral load testing is not available. Hence, ART initiation and monitoring is based on the WHO clinico-immunological approach. However, as this approach lacks viral load determination and drug resistance monitoring by sequencing of reverse transcriptase gene segments HIV infected adults and children may be at risk for “unrecognized” virologic failure and the subsequent development of antiretroviral drug resistance. The aim of this study was to evaluate the virological efficacy and CD4^+^ T cell recovery among HIV/AIDS adults and children patients who were on ART for up to 6 years.

## Methods

### Patients and ART

Patients were assessed at Gondar University hospital (GUH), Northwest Ethiopia. GUH is a referral and teaching hospital responsible for the treatment of more than 8, 000 HIV infected adults and 400 children. Adult patients with WHO clinical stage 4 irrespective of CD4^+^ T cell count, stage 3 with CD4^+^ T cell count ≤ 350 cells/mm^3^, or patients with CD4^+^ T cell count ≤ 200 cell/mm^3^ in any clinical stages are eligible for ART [22]. HIV-infected children with WHO clinical stage III or IV disease (regardless of CD4 cell count) or children with CD4 percentage < 20% (for children < 18 months) or <15% (for children >18 months), regardless of clinical stage, were eligible to initiate ART [21]. However, the adult’s treatment protocol is revised since 2010 according to WHO recommendation for resource limiting settings towards initiation of ART at CD4^+^ T count of 350 cells/mm^3^[23]. Similarly, all infants <12 months with WHO clinical stage III or IV disease (regardless of CD4 cell count) or children with CD4 percentage <20% (for children <36 months) or <15% (for children aged > 36 months), regardless of clinical stage, were eligible to initiate ART [22]. ART in Ethiopia consists of generic low-cost fixed-dose combination (FDC) of two NRTI and one NNRTI with first line regimens of lamivudine (3TC) combined with stavudine (d4T) or zidovudine (AZT), and either nevirapine (NVP) or efavirenz (EFV) (Table 1). For anaemic patients d4T substitutes AZT and for tuberculosis patients treated with rifampicin NVP replaces EFV. Co-trimoxazole prophylaxis was given to all patients included in this study. ART and care was free of charge and there were no interruptions in drug supply. In the event of first-line treatment failure, second-line regimens with addition of lopinavir/retrovir were started [21,22]. Other antiretroviral drugs such as protease, integrase and entry inhibitors as well as co-receptor antagonists are not in use. Both diagnosis of first-line treatment failure and criteria for switching to second-line drugs is based on clinical findings and CD4^+^ T cell count as viral load is not routinely measured.

**Table 1:** Baseline characteristics of adult HIV/AIDS patients on ART at GUH

		**Number of patients**	
**Variables**		**Adults**	**Children**
WHO clinical stage			
	I	3	4
	II	11	6
	III	72	57
	IV	14	33
Anaemia status (%)^a^			
	Anaemic	62	67
	Non anaemic	38	33
CD4 cell count (cells/mm^3^)^b^			
	<50	6	0
	50-99	25	5
	100-199	55	14
	200-349	14	33
	≥350	0	48
Total duration on ART (months)			
	13-24	100	100
	25-36	47	56
	37-48	13	23
	49-60	4	15
	≥61	0	5
ART regimen			
	1a: 3TC + d4T + NVP	39	57
	1b: 3TC + d4T + EFV	32	5
	1c: 3TC + AZT** + NVP	17	35
	1d: 3TC + AZT + EFV***	12	3

Keys: 3TC (Lamivudine), d4T (Stavidine), NVP (Nevirapine), EFV (Efavirenz), AZT (Zidovidine); The difference in ART regimen between adults and children is due to the high rates of Tuberculosis in adults; **AZT replaced by d4T in anaemic patients; *** EFV replaced by NVP in patients with TB when on rifampicin;^*a*^*Age and gender specific classification of anaemia according to WHO (*http://www.who.int/vmnis/indicators/haemoglobin*);*^*b*^*Age specific classification for ART eligibility.*

### Study design and selection procedure

This longitudinal study follows the WHO protocol for assessment of acquired drug resistance for adults [24]. Consecutive HIV-infected adult (N = 100) and children (N = 100) who had received first-line ART for more than 12 months and visiting Gondar Hospital from June to November 2008 were included in the study. Patients who had interrupted treatment or patients transferred from another ART clinic or pregnant women or those with known chronic illness were excluded.

### Clinical and laboratory assessment

Minimal socio-demographic data and relevant clinical features of the patients were retrieved from medical records. About 10 ml venous blood was collected in a tube containing ethylene diamine tetra-acetic acid (EDTA). After centrifugation (956 rcf for 5 minutes) plasma was separated and stored at -40°C until further used. CD4^+^ T cell count was done at baseline and at 6 months interval using the FACSCount flow cytometer (Becton Dickinson, San Jose, CA, USA) following the manufacturer’s protocol. The laboratory is accredited by the American Society of Clinical Pathologists and participated in external quality control and assurance program.

### RNA extraction and plasma viral load determination

RNA was extracted from 0.6 ml of plasma with the Abbott *m*2000*sp* automated sample preparation system (Abbott Molecular, Des Plaines, IL, USA) according to manufacturer’s instructions. Plasma viral load was measured using Quantitative RealTi*m*e HIV-1 assay by the Abbott *m*2000*rt* instrument with a lower detection limit of 40 copies/ml.

### Sequence determination of HIV-1 pol gene

Viral RNA was reverse transcribed using AMV reverse transcriptase (Promega Corporation, WI, USA) and the outer primer HIVrt (5′TGTTTTACATCATTAGTGTG 3′). The entire protease (PR) and partial (76%) reverse transcriptase (RT) regions of the *pol* gene were amplified using an in house assay. In brief, Phusion Hot Start High-Fidelity DNA polymerase (Finnzymes, Espoo, Finland) was used in nested PCR with the outer primers HIVpcrFor1 (5′TGATGACAGCATGTCAGGGAGTGG3′) and HIVpcrRev1 (5′GGCTCTTGATAAATTTGATATGTCCATTG3′) yielding a 1757 bp amplicon, and subsequently by the inner primers HIVpcrFor2 (5′AGCCAACAGCCCCACCAG3′) and HIVpcrRev2 (5′CTGTATTTCTGCTATTAAGTCTTTTG 3′) yielding a 1389 bp amplicon. Initial denaturation was done at 98°C for 2 minutes followed by 40 cycles consisting of 10 seconds of denaturation at 98°C and 25 seconds of annealing at 64°C for the first round outer primers (HIVpcrFor1 and HIVpcrRev1) PCR and at 53°C for the nested inner primers (HIVpcrFor2 and HIVpcrRev2) PCR with a 40 seconds extension at 72°C for both and final extension for 5 min at 72°C. Purified PCR products were subjected to direct sequencing of both the sense and antisense strands using Big Dye Terminator Cycle Sequencing Ready Reaction kit (Applied Biosystems, Foster City, CA, USA). For each sample, six separate sequencing reactions were done using the two inner PCR primers and four additional internal primers: HIVseq1 (5′GTTAAACAATGGCCATTGACAGA3′), HIVseq2 (5′TGGAAAGGATCACCAGCAATATT3′), HIVseq3 (5′GGGCCATCCATTCCTGGCT3′) and HIVseq4 (5′2CCATCCCTGTGGAAGCACATT3′) which allowed a double coverage of the *pol* region. All primers positions are matched to HIV-1HXB2 (GenBank accession number K03455). Both forward and reverse overlapping sequences were manually edited with the Geneious Basic software version 5.4 [25]. Genotypic drug resistance was defined according to The Stanford University HIV Drug-Resistance Database (http://hivdb.stanford.edu/).

### Statistical analysis

The main outcomes of interest were virological suppression (HIV RNA < 40 copies/ml), drug resistance mutation/s and immunological recovery. Virological suppression was defined as HIV viral load <40 copies/ml. Immunological recovery was evaluated based on CD4^+^ T cell response: patients who failed to achieve an absolute increase in CD4^+^ T cell count from baseline by at least 50 cell/mm^3^ at 12 months were defined as immunological non responders. Those patients who achieved an absolute CD4^+^ T cell count of 200 cells/mm^3^ at the 12 months visit were defined as immunological responders. Absolute response in CD4^+^ T cell count was calculated at every 6 months intervals and categorized into 2 phases: Phase I from base line to 12 months, Phase II from 13–48 months. Duration of ART was rounded to the nearest half or full year. Univariate analysis was performed for sex, age, WHO clinical stages, ART regimen at baseline, duration of ART, haematocrit value and CD4^+^ T cell count. Logistic regression was used to study associations between baseline characteristics and outcomes. A p-value of less than 0.05 was considered statistically significant. The statistical analyses were carried out using SPSS statistical software version 17.

### Ethical issues

The work meets relevant ethical guidelines. Institutional ethical clearance was obtained from the University of Gondar Ethics Review Committee. Written and/or verbal informed consent was also obtained from study subjects and/or families and/or guardians.

## Results

### Baseline patient’s characteristics

A total of 200 HIV-infected patients (100 adults and 100 children) were included in the present study with the mean ± standard deviation age of 34.2 ± 8.4 and 5.3 ± 1.2 years, respectively. At baseline the median CD4^+^ T cell count for adults and children was 144 cells/mm^3^ (Inter quartile range (IQR) 91.25-181.75) and 345 (IQR 17–1435). The median duration with ART was 24 months (IQR: 18.00-29.25) for both adults and children. The proportions of patients with CD4^+^ T cell strata, WHO clinical stages, the duration of time on ART, the main therapeutic regimen are summarized in Table 1. A standard first-line ART regimen as defined by national ART guidelines was initiated: 3TC + d4T + NVP for 39% of adults and children; 3TC + AZT + NVP for 12 and 57% of adults and children (Table 1). All the children except two were born from HIV infected mothers without exposure to single dose nevirapine (sdNVP) and combined ART for prevention of mother to child transmission (PMTCT) prophylaxis.

### Virological response to ART

Virological suppression was observed in 82% of the adults in median time of 24 months on ART. The proportion of the adult patients with plasma HIV RNA level of 41–400 copies/ml, 401–1000 copies/ml and >10,000 copies/ml were 14, 2 and 2, respectively. There was no significant difference in the proportion of patients with baseline CD4^+^ T cell counts of 50–99 cells/ml (OR 2.8; 95% CI 0.143-55.56), 100–199 cell/mm^3^ (OR 3.6; 95% CI 0.35-32.32) or with baseline CD4^+^ T cell counts of >200 cells/mm^3^ (OR 3.23; 95% CI 0.38-27.34) who achieved a viral load of <40 copies/ ml as compared with those with a baseline CD4^+^ T count of < 50 cells/mm^3^. Samples with plasma viral load of >400 copies/ml were amplified and genotyping was successful in 4 patients and all were found to be HIV-1 subtype C. Mutation K103N conferring resistance to the NNRTIs NVP, delavirdine (DLV), EFV and etravirine (ETR) was found in one patient. Polymorphic accessory mutation to the protease inhibitor class of ARVs at codon 74 (T74S) was observed in 2 patients. In 3 patients, naturally occurring polymorphisms that may or may not have an impact on levels of drug resistance were found (mutations M36I, H69K and L89M) (Table 2). After additional 24 months of ART, patients were retrieved. Although the attrition rate was high, 22 patients were found still on ART and all of them were virological suppressed.

**Table 2:** **Genotyping drug resistance mutations in patients with ART failure**^**a**^

**Code**	**ART regimen**^**b**^	**Time on ART**	**PVL**^**c**^		**Mutations**	**Subtype**
		**(months)**		**PR**	**RT**	
0053	1a/1c	40	13945	M36I, H69K, T74S, L89M	-	C
1959	1c	26	598	M36I, H69K, T74S, L89M	-	C
3438	1d	13	495	M36I, H69K, L89M	-	C
1739	1b	25	18239	M36I, H69K, L89M	K103N	C
2369	1c	19	25678	M36I, H69K, L89M	M41L, L210W	C

^*a*^*ART failure was defined as plasma viral load of > 400 copies/ml;*^*b*^*: As indicated in Table*1*; *^*c*^*PVL: Plasma viral load in copies/ml.*

Similar to the adults, high virological suppression rate (87%) was observed in children in a median time of 24 months on ART. One HIV-1 subtype C isolate with thymidine analogue mutations (M41L, L210W) was detected. Low level viraemia (HIV-1 RNA in the range of 41–1000 copies/ml) was observed in a single sample of 13 clinically asymptomatic children.

### CD4^+^ T cell response to ART

**Figure 1:**
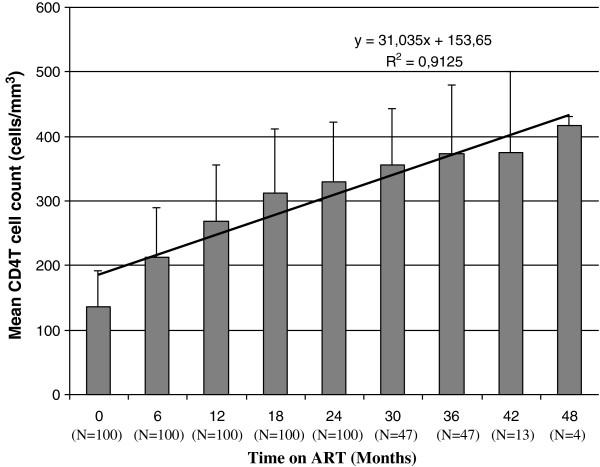
**CD4**^**+ **^**T cell count over a period of 48 months.** Trend in CD4^+^ T cell recovery among HIV/AIDS adult patients over a period of 48 months on ART. The straight line in the graph is the trend line showing the increase of CD4^+^ T cells at a steady rate over 48 months on ART. The R-squared value is 0.9125 shows a good fit of the line to the data. The equation (Y = 31.35X + 153.65) on the graph is the regression analysis that extends a trend line in a chart beyond the actual data to predict future values.

The proportion of adult HIV infected patients with CD4^+^ T cell counts <100 cells/mm^3^ was 31% at baseline. This proportion had decreased to 6% by month 12 of the treatment. The median CD4^+^ T cell count increased nearly two-fold from baseline, reaching 266 cells/mm^3^ after 12 months on ART (Figure 1). It further increased reaching 336, 397 and 422 cells/mm^3^ after 24, 36 and 48 months of ART, respectively. The rate of CD4^+^ T cell count increase in the first 12 months period was higher than the rates in both the 13–24 months and 25–48 months intervals (P < 0.001, 95% CI, 63.27-88.01). Thus, the pattern of CD4^+^ T cell count increase on average in 6 months time, and in the next 7–12 months was 75.64 cells/mm^3^ and 58.8 cell/mm^3^, respectively, with statistically significant mean difference (P = 0.023). However, the further increase in the ensuing 12 months of ART was slower with a mean of 47.33 cells/mm^3^ at 13–18 months and 3 cells/mm^3^ at 19–24 months and did not reach at statistical significance level. Accordingly, the rate of CD4^+^ T cell count increase was divided into 2 phases: a rapid phase (0–12 months; mean = 11.5 cells/mm^3^/month), and a slower phase (13–48 months; mean = 3 cells/mm^3^/month) (Figure 1).

In a similar trend, the median absolute CD4^+^ T cell count of children has increased from 345 (IQR 17 to1435) to 998 (IQR 678–2205) in a median time of 12 months on ART. The CD4^+^ T cell counts after a median time of 24 months on ART were similar between viraemic (n = 13) and aviraemic (n = 87) children (median (IQR) 996 (730–1524) versus 946 (472–1102) (P = 0.10) cell/mm^3^.

### Effect of baseline CD4^+^ T cell count and ART regimens on rates of CD4^+^ T cell increase

**Figure 2:**
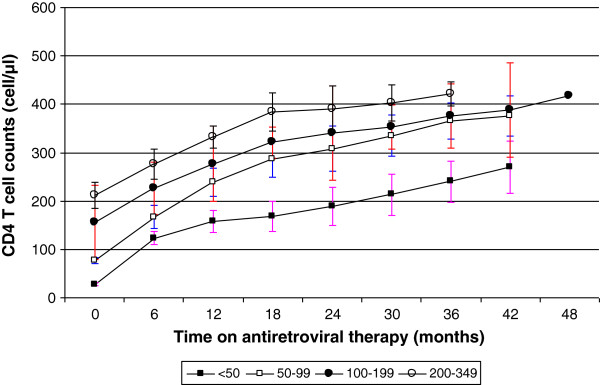
**Mean CD4**^**+ **^**T cell recovery in different strata during ART.** The mean CD4^+^ T cell recovery of adult patients with low baseline CD4^+^ T cell strata (< 50 cells/mm^3^) and higher CD4^+^ T (200–349 cells/mm^3)^ at the initiation of ART over a period of 48 months on ART.

Adult patients with low baseline CD4^+^ T cell count (i.e. < 50 cells/mm^3^) at the initiation of ART required more time to recover compared to those patients with a higher baseline CD4^+^ T cell count (Figure 2). In the 48 months period, none of the patients reached a final CD4^+^ T cell count of 500 cells/mm^3^. Patients with lowest CD4^+^ T cell count strata (<50 cells/mm^3^) did not reach the lower threshold of 350 cells/mm^3^ up to 42 months on ART, though there was a remarkable increase to an average of 276 cells/mm^3^ (Figure 2). However, patients with average baseline CD4^+^ T cell count strata 50–99, 100–199 and 200–349 attained 350 cells/mm^3^ by 33, 28, and 14 months after ART, respectively. Among those who had a CD4^+^ T cell count measurement at 12 months (n = 96), the CD4^+^ T cell count increased by >50 cells/mm^3^ in 16 (16.7%) patients. Of these, the viral load was suppressed below LLD- lower limit of detection (<40 copies/ml) in 13 patients in a median time of 24 months on ART. Among patients with baseline CD4^+^ T cell counts of <50, 50–99, 100–199 and ≥ 200 cells/mm^3^, the proportions of patients who were immunological non-responders were 1, 1, 10 and 4, (16%, 4%, 20%, 30%) respectively. Failure to attain 200 cells/mm^3^ was not significantly associated (Table 3) with older age, or lower baseline CD4^+^ T cell count (P > 0.05 in all comparisons). The recovery of CD4^+^ T cell count follows similar pattern independent of the antiretroviral drug regimen up to 24 months in both adults and children. However, patients with ART regimen 1c and 1d showed a slight increment in CD4^+^ T count between 24 and 42 months of ART. During the study period, there was a treatment switching in 15 patients due to peripheral neuropathy secondary to d4T associated toxicity (n = 7), AZT induced anaemia (n = 2) and d4T related lipoathrophy (n = 1). The incidence of tuberculosis during the first 6 months on ART was 7% and hence NVP was substituted by EFV in those patients.

**Table 3:** **Logistic regression models predicting overall change in CD4**^**+ **^**T cell count of adults during ART**

**Variables**		^**a**^**Risk of non-response**		^**b**^**Risk of failure to attain >200 CD4 cell**	
		**OR (95% CI)**	**P value**	**OR (95% CI)**	**P value**
Sex	Female	0.2 (0.65-0.658)	0.08	6.5	
	Male	0.4 (0.16-1.33)	0.150		
Age (years)	<29		0.76		0.52
	30-39	1.68 (0.401-7.09)	0.47	14.0 (1.62-122.7)	0.160
	≥40	1.17 (0.32-4.21)	0.81	1.88 (0.60-5.90)	0.278
WHO Clinical Stage I	I		0.69		0.4
	II	0.154 (0.007-3.57)	0.244	0.038 (0.002-0.894)	0.045
	III	0.346 (0.027-4.418	0.414	0.769 (0.043-13.866)	0.8
	IV	0.359 (0.043-3.013)	0.345	0.325 (0.039-2.7)	0.32
Haematocrit value	< 37%	3.0		3.0	
	≥ 37%	2.54 (0.848-7.514)	0.096	2.16 (0.751-6.253)	0.153
ART regimen					
	3TC + d4T + NVP	1.66 (0.349-7.9)	0.542	4.857 (0.974-24.227)	0.054
	3TC + d4T + EFV	1.87 (0.36-9.64)	0.450	2.286 (0.50-10.44)	0.286
	3TC + AZT + NVP	6.00 (0.53-67.27)	0.146	2.667 (0.463-15.35)	0.272
	3TC + AZT + EFV		0.54		0.288
CD4^+^ T cell count	<50		0.313		0.99
	50-99	2.00 (0.174-22.94)	0.578	0.578 0.99 (0.00-)	0.9
	100-199	9.20 (0.91-93.02)	0.060	0.060 0.99 (0.00-)	0.9
	200-349	1.68 (0.436-6.47)	0.451	0.451 0.99 (0.00-)	0.9

^*a*^*Risk of immunological non-response (an increase of <50 cells/mm*^*3*^*at 12 months).*

^*b*^*Failure to attain an absolute CD4*^*+ *^*cell count of ≥ 200 cells/mm*^*3 *^*after 48 weeks ART).*

## Discussion

The 82% suppression rates and the low level of HIV drug resistance in adults with a median time of 24 months on first line ART in the present study from Ethiopia demonstrates a high level of effectiveness of the antiretroviral agents in the setting. The result is in agreement with data from Europe and North America [9] and with a more recent report from Tanzania [26] where 88% viral suppression (defined as viral load below 400 copies/ml) with a median follow up time of 23 months has been described. A good short term virological efficacy rates have been reported and a systematic review on virological outcomes after 12 months ART from low-to-middle-income countries where 83-86% of virological efficacy on treatment was reported [27]. The high virological suppression rate of 87% with low-level drug resistance among the paediatric age groups of this study is somewhat unexpected too but is similar to a recent study from Mozambique [28]. On the other hand, the detection of low level viraemia (13%) and thymidine analogue mutation (1%) in children documented to receive PMTCT prophylaxis is expected. The viral suppression rates in both adult and children for up to four years after starting first line ART in a setting where nearly half of the population living under poverty line are affected by various co-morbid infectious diseases and where access to ART is largely restricted to two drug classes is encouraging. The results exceeded the WHO suggested target of ≥70% [24] and are similar to reports from other settings [19,20,26-28].

The high rate of virological suppression may have an impact on HIV prevention and reducing the overall risk of transmission at a population level. It has been suggested that scaling up of ART results in a level of virological suppression at the population level that will reduce HIV transmission [29-32]. To reach this goal ART should be extended to WHO groups I and II. However, the transient viraemia observed in this study among both adults and children of clinically asymptomatic conditions could be an early warning indicator for early virological failure as some may suppress and others may develop virological failure if tested in future. It does not appear to affect CD4^+^ or CD8^+^ T-cell counts or the risk of subsequent virological failure. Natural variation, assay effects and adherence might all have a role.

The sustained virological efficacy on 6 years ART in the present study might be attributed to free provision of ART and care for HIV infected patients as it has been previously shown to improve treatment efficacy [9] and the pre and post ART provision readiness and adherence counselling [8]. Moreover, it might have been related to the strong collaboration between the nurses and the community based workers that ensured a follow up of patients and their network at village and home level. In addition, because traditional medicine is culturally entrenched, accessible, and affordable, up to 80% of the Ethiopian population relies on traditional remedies (herbs) as a primary source of health care [33]. This is also the case for treatment of AIDS, but its contribution to treatment success has not been evaluated and certainly not been demonstrated yet. Nevertheless, it is claimed that certain medicinal plant remedies improve the quality of life of patients with AIDS by reducing the viral load [34].

It has been documented in European patients that, after the initiation of ART, peripheral CD4^+^ T cell count starts rising and continues so for at least 3–5 years [35]. Our data indicate a rapid initial increase in CD4^+^ T cell count in the first 6 months and in the following 7–12 months by 12.2 cells/mm^3^/month and 9.6 cell/mm^3^/month, respectively, followed by almost linear rise in the subsequent two years. This is in agreement with a report that shows the rapid initial increase in CD4^+^ T cell count in the first 6 months [36] which relies on a reduction in T-cell activation and primarily consists of a release of memory CD4^+^ T cells trapped in the lymphoid tissue. However, the relative slow increase rate of CD4^+^ T cell after 12 months of ART (13–24 months: mean = 6.3 cells/mm^3^/month; 25–48 months: mean = 3 cells/mm^3^/month) and the gradual annual changes in CD4^+^ T cell count thereafter suggests that the number of CD4^+^ T cell may reach a plateau level sometime in the future. In this case the naïve CD4^+^ T-lymphocytes from the thymus, as well as memory CD4^+^ T-lymphocytes, contribute to the reconstitution of the immune system [37]. The data also shows that those patients with baseline CD4^+^ T cell count of <50 cells/mm^3^ had similar rate of increase in the first 6 months and the consecutive months compared with higher baseline CD4^+^ T cell count. In most HIV-1 infected individuals treated with ART, CD4^+^ T cell recover to levels above 500 cells/mm^3^, at which HIV-1 related clinical complications are rare [36]. None of the patients in this study reached this threshold in 48 months of ART which could be associated with the naturally lower CD4^+^ T cell counts in adult Ethiopians irrespective of HIV infection [38,39] and lower immune recovery of Africans [40]. The relative high CD4^+^ T cell counts and optimal CD4^+^ T cell recovery among children in the current study is parallel with the data that shows the predominance of naive CD4^+^ T cell phenotype at birth which is comparable to Caucasians [41].

## Conclusions

After a median time of 24 months on ART, high viral suppression rates in adults (82%) and children (87%) were observed in Gondar hospital, Northwest Ethiopia where patients usually present late in the course of infection. This suggests that antiretroviral drugs in the setting can sustain virological efficacy for a substantial length of time and enhance immunological recovery irrespective of age. However, the presence of drug resistance mutations and low level viraemia among clinically asymptomatic patients highlights the need for regular virological monitoring in order to optimise treatment success and preserve future treatment options.

## Competing interests

The authors have no competing interests to declare.

## Authors’ contributions

AM: study design, data collection, analysis and interpretation and draft the manuscript; UGL: study design, data interpretation and revising the manuscript; MM: study design, data analysis and interpretation and revising the manuscript. All authors have approved this final version for submission.

## Pre-publication history

The pre-publication history for this paper can be accessed here:

http://www.biomedcentral.com/1471-2334/14/28/prepub
